# Emerging roles of fibroblast growth factor 21 in critical disease

**DOI:** 10.3389/fcvm.2022.1053997

**Published:** 2022-11-10

**Authors:** Fang Yan, Li Yuan, Fan Yang, Guicheng Wu, Xiaobo Jiang

**Affiliations:** ^1^Department of Geriatrics, Chengdu Fifth People’s Hospital, Geriatric Diseases Institute of Chengdu, Chengdu, China; ^2^Center for Medicine Research and Translation, Chengdu Fifth People’s Hospital, Chengdu, China; ^3^Department of Clinical Laboratory Medicine, Chengdu Fifth People’s Hospital, Chengdu, China; ^4^Department of Endocrinology, Chengdu Fifth People’s Hospital, Chengdu, China; ^5^Department of Hepatology, School of Medicine, Chongqing Municipality Clinical Research Center for Endocrinology and Metabolic Diseases, Chongqing University Three Goreges Hosipital, Chongqing University, Chongqing, China; ^6^Department of Cardiology, Chengdu Fifth People’s Hospital, Chengdu, China

**Keywords:** fibroblast growth factor 21, acute lung injury, acute respiratory distress syndrome, acute myocardial injury, acute kidney injury, sepsis

## Abstract

In spite of the great progress in the management of critical diseases in recent years, its associated prevalence and mortality of multiple organ failure still remain high. As an endocrine hormone, fibroblast growth factor 21 (FGF21) functions to maintain homeostasis in the whole body. Recent studies have proved that FGF21 has promising potential effects in critical diseases. FGF21 has also been found to have a close relationship with the progression of critical diseases and has a great predictive function for organ failure. The level of FGF21 was elevated in both mouse models and human patients with sepsis or other critical illnesses. Moreover, it is a promising biomarker and has certain therapeutic roles in some critical diseases. We focus on the emerging roles of FGF21 and its potential effects in critical diseases including acute lung injury/acute respiratory distress syndrome (ALI/ARDS), acute myocardial injury (AMI), acute kidney injury (AKI), sepsis, and liver failure in this review. FGF21 has high application value and is worth further studying. Focusing on FGF21 may provide a new perspective for the management of the critical diseases.

## Introduction

Despite the great progress that has been made in the management of critical diseases in recent years, multiple organ failure is still associated with a high mortality rate (1). Thus, the care for critically ill patients is becoming increasingly complex, and several fundamental problems remain (2). One of which is the lack of biomarkers with sufficient sensitivity and specificity for critical illnesses, although some progress has been made in the recent two decades (3, 4). The new molecules and serologic markers that have been discovered for the clinical management of critical illnesses are restricted by many factors (5–7). The foremost problem is the challenge of applying biomarkers with speed and accuracy during routine clinical care. Furthermore, the benefit of markers can be limited due to the lack of specific clinical interventions/strategies for many critical diseases, which are mostly treated with supportive therapies.

Fibroblast growth factor 21 (FGF21) is an endocrine factor that is synthesized predominantly by the liver and has integral function to the maintenance of energy homeostasis (8). It is a member of the FGF19 subfamily, which is different from other typical FGF members (9). The focus of previous research on this protein has focused on its roles relating to energy, glucose/lipid metabolism, and insulin sensitivity (10–15). Both animal and population studies have provided insights into this field and demonstrated that FGF21 in circulation is elevated in several diseases and can be served as a potential biomarker (16–19). FGF21 is also closely involved in the progression of acute-on-chronic liver failure (ACLF) and has a great predictive potential for organ failure (20–22). Hence, we aim to review and summarize the current status of FGF21 research in critical illness, including acute lung injury (ALI), acute respiratory distress syndrome (ARDS), acute myocardial injury (AMI), acute kidney injury (AKI), sepsis, and liver failure.

## Biogenesis, characterization, and functions of fibroblast growth factor 21

The *FGF21* resides on human chromosome 19 (19q13.33) and is comprised of three exons (23). The FGF21 is composed of 209 amino acids, of which 29 amino acids at the N-terminus form a signaling peptide (24). The FGF21 and Fgf21 (human/mouse) amino acid sequences are 75% conserved between the two species (24). FGF21 is not only primarily synthesized and secreted by the liver but also highly expressed in the pancreas, adipose tissue, thymus, and endothelial cells (25–29). Hepatic *FGF2*1 expression is affected by starvation signaling and conditions that cause inflammation and illness (8, 30, 31). During fasting, *FGF21* expression is upregulated in the liver by activating the peroxisome proliferator-activated receptor α (PPARα) and retinoic acid receptor (RXR) heterodimer, and glucagon-stimulated protein kinase (PKA) (32, 33). Additionally, glucose can induce hepatic *FGF21* expression as a satiety signal by activating carbohydrate-response element-binding protein (ChREBP). Moreover, hepatic *FGF21* expression has been proven to be induced by diet in a murine model, and *via* a PPARα agonist (33, 34). Similarly, FGF21 concentrations were increased in patients treated with the PPARα agonist, fenofibrate (25). FGF21 synthesized in the liver can be secreted into the blood as a major source of circulating FGF21 in serum, which plays a role throughout the body. However, in adipocytes, *FGF21* expression is regulated by PPARγ/RXR heterodimer and ChREBP (35, 36). The FGF21 synthesized during adipose tissue functions (autocrine or paracrine) cannot enter into the circulation to exert endocrine effects, which can enhance PPARγ functions (37, 38), so it will not affect the level of FGF21 in the blood. Pretreatments with the PPARγ antagonist have been shown to partially abrogate FGF21-induced adiponectin secretion and significantly reduce FGF21-induced adiponectin mRNA expression in mouse adipocytes (39).

**FIGURE 1:**
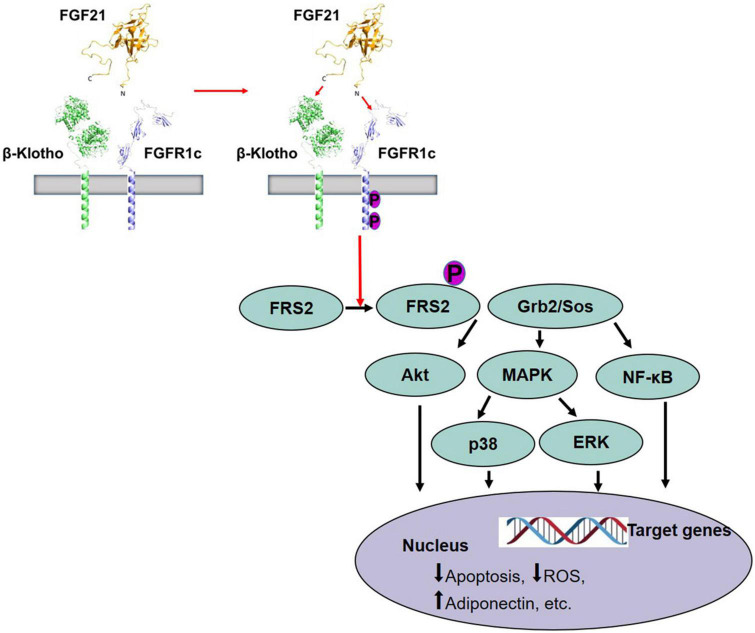
Activation of FGFR1c by FGF21 and signaling pathway (42). FGF21 receptor consists of FGFR1c and co-receptor β-Klotho that are constitutively associated with the plasma membrane. The C-terminus of FGF21 interacts with β-Klotho, and β-Klotho is necessary for FGF21 to perform the function. Then, the N-terminus of FGF21 binds to FGFR to form a stable FGF21/β-Klotho/FGFR complex and leads to autophosphorylation of FGFR1, and phosphorylation of FRS2, which then forms a complex with Grb2/Sos to activate downstream molecular signals, such as Akt pathway, MAPK/p38 pathway, MAPK/ERK pathway, and NF-κB pathway, causing the change of target genes in the nucleus. FGF21, fibroblast growth factor 21; FGFR, FGF receptor; Akt, protein kinase B; MAPK, mitogen-activated protein kinase; ERK, extracellular signal-regulated kinase after FGF receptor.

The molecular mechanisms of FGF21 signaling action have been comprehensively explored. FGF21 lacks a heparin-binding domain, which is required to bind to Klotho family transmembrane proteins and form a common complex to activate FGF receptor (FGFR) signaling, instead, forming a signaling complex with β-Klotho and FGFR (40, 41). The FGF21 N- and C-terminal regions are both closely related to their biological activity, yet perform different functions. Namely, FGF21 C-terminus interacts with β-Klotho, which is required for FGF21 to regulate blood glucose and lipid metabolism, whereas the N-terminus binds to FGFR to constitute an FGF21/β-Klotho/FGFR complex thereby activating downstream molecular signals to exert its biological effects (42). Thus, FGF21 performs specific functions *via* β-Klotho as part of a non-activated FGFR-mediated signaling pathway (Figure 1). Recent studies have also confirmed that FGF21 binds to FGFR1c, FGFR3c, and FGFR4, the former being the predominant receptor (40, 41). The specific expression of β-Klotho and FGFR subtypes within tissues determines the specificity of FGF21 and its functional implications. β-Klotho has been confirmed to be an essential co-receptor for FGF19 and FGF21 that is not only specifically expressed in the liver but is also abundantly expressed in several other tissues, such as the central nervous system (CNS), pancreatic islets, and adipose tissue (43–47). The outer domain of typical FGFRs contains three immunoglobulin domains (D1–D3). The D1 and the D1–D2 linker (D1/linker region) of FGFR1c inhibit interactions with β-Klotho (40), which can be overcome *via* FGF21 C-terminal binding (46).

Besides regulating glucose and lipid utilization, FGF21 also regulates insulin sensitivity and ketogenesis (48–50). It has been reported that FGF21 significantly enhanced glucose uptake in 3T3-L1 adipocytes through upregulation expression of GLUT1 for the first time in 2005 (51). In addition, FGF21 participates in regulating glucose uptake in primary myotubes, myoblast cells, and the heart (40, 52). FGF21 is also a major lipid metabolism regulator in various tissues and can significantly reduce cholesterol, low-density lipoprotein, and triglycerides and increase adiponectin and high-density lipoprotein levels in plasma (53). It is interesting to note that FGF21 manifests anti-inflammatory properties in the pancreas, the heart, and the skeletal muscles (54, 55). Additionally, FGF21 inhibits apoptosis in endothelial and cardiomyocytes (56, 57). For reducing the apoptosis of myocardial cells, FGF21 also alleviates antagonism against the effects of ischemia/reperfusion (I/R) and oxidative stress and protects the heart effectively (58). FGF21 is involved in main biological processions in human beings, and some clinical trials provided an initial advancement for human FGF21 analogs as a worthy therapeutic candidate in type 2 diabetes mellitus (T2DM), non-alcoholic fatty liver disease (NAFLD), and non-alcoholic steatohepatitis (NASH) (Table 1). However, therapeutic studies of FGF21 in critical diseases in human populations are not yet available. More studies are needed to elucidate the mechanism to expand its clinical practice and use.

**TABLE 1:** Clinical trials of human FGF21 analogs in human beings.

Name	Structure feature	Condition or disease	Administration route	Dosage	References	Study results
LY2405319	Modified human FGF21	T2DM	Subcutaneous injection	3, 10, or 20 mg daily for 28 days	Gaich et al. (98)	Reducing blood glucose, insulin and body weight
PF-05231023	Two FGF21 joint with an IgG1 backbone	T2DM	Intravenous infusion	25, 50, 100, 150 mg weekly for 4 weeks	Kim et al. (99)	Lowering triglycerides markedly in the absence of weight loss, with modest changes in markers of bone homeostasis
BMS-986036 (pegbelfermin)	Peglylated human FGF21	T2DM	Subcutaneous injection	1, 5, 20 mg daily for 12 weeks	Charles et al. (100)	Improving high-density lipoprotein cholesterol and triglyceride.
BMS-986036 (pegbelfermin)	Peglylated human FGF21	Liver fibrosis, NAFLD, NASH	Subcutaneous injection	10, 20, or 40 mg weekly for 48 weeks	Abdelmalek et al. (101)	Reducing hepatic fat and improving metabolic factors and biomarkers of hepatic injury and fibrosis
AKR-001 (efruxifermin)	Fc-FGF21 engineered fusion protein	NASH	Subcutaneous injection	28, 50, 70 mg weekly for 16 weeks	Harrison et al. (102)	Reducing of serum non-HDL-C, triglycerides, ALT, and GGT

ALT, alanine aminotransferase; GGT, gamma-glutamyl transferase; NAFLD, non-alcoholic fatty liver disease; NASH, non-alcoholic steatohepatitis; non-HDL, non-high density lipoprotein cholesterol; T2DM, type 2 diabetes mellitus.

## The role of fibroblast growth factor 21 in acute lung injury and acute respiratory distress syndrome

Acute lung injury and its worst form ARDS are the main causes of acute respiratory failure. Therefore, they constitute the main reason for death in patients with the intensive care unit (ICU) (59).

In the current research of medicine, the studies investigating the association between FGF21 and ALI/ARDS have been limited. Li et al. (17) demonstrated that serum FGF21 levels were elevated in patients with sepsis and ARDS after ICU admission, and the risk of 28-day mortality was strongly associated with FGF21. Similarly, FGF21 reduced inflammatory response and apoptosis so that rescued lipopolysaccharide (LPS)-induced ALI that was induced by LPS through the classic inflammatory signaling pathways of TLR4/MyD88/nuclear factor kB (NF-κB) (60). Based on the recent evidence, FGF21 might be a new effective method for the treatment of ALI (60), and further studies are required to investigate the underlying mechanisms and explore the potential clinical use of FGF21 for lung disease in the future.

## The role of fibroblast growth factor 21 in acute myocardial injury

Cardiovascular diseases are the main issues of public health in modern society. AMI is the most serious disease of the coronary artery because it is the major factor in most death cases in the world (61). In addition, AMI decreases cardiac function and remains the commonest cause of heart failure. Therefore, it is essential to understand the pathogenesis of cardiac remodeling.

The FGF21 has great importance in the process of ventricular remodeling and may be related to coronary artery disease (62) and diastolic heart failure. In the patients with AMI, the expressions of FGF21 were significantly increased in the first 24 h after myocardial infarction and maintained a high level for 1 week (63). Furthermore, FGF21 was related to the expression of brain natriuretic protein and strongly predicted adverse events of the cardiovascular system in ST-segment elevation myocardial infarction patients after percutaneous transluminal coronary intervention (64). Thus, serum FGF21 may be a potential biomarker in cardiac diseases (63). Administration of FGF21 decreased the mRNA expression of interleukin-6 (IL-6) and tumor necrosis factor-α (TNF-α) and protected against pathological myocardial remodeling and improved cardiac function at 2 weeks in a myocardial infarction mouse model (65). In a cardiac hypertrophy model, the lack of FGF21 increased cardiac reactive oxygen species (ROS) accumulation (66). These cardiac protections of FGF21 may be involved with multiple intracellular signaling pathways, which include phosphatidylinositide 3-kinase (PI3K) –protein kinase B (Akt), p38 mitogen-activated protein kinase (MAPK), and extracellular signal-regulated kinase 1/2(ERK1/2) (67, 68). For example, upregulation of FGF21 may reduce myocardial infarction size after I/R injury through FGFR1–PI3K–AKT pathway (67).

The FGF21 is related to cAMP-response element binding protein-eroxisome proliferator-activated receptor-gamma coactivator 1 alpha (CREB-PGC-1a) pathway, and it leads to an induction of anti-oxidant genes, inflammatory response, and suppression of pro-apoptotic proteins. Thus, it may have a significant impact on I/R injury (69). FGF21 protects cardiomyocytes from I/R injury by promoting the elevation of mir-145 and autophagy, providing a new protection cardiomyocyte strategy (70). Interestingly, FGF21 elevated by exercise training has anti-cardiac fibrosis by the inactivation of the TGF-β1—mad2/3–MMP2/9 signaling pathway (71). More importantly, FGF21 regulated energy homeostasis and mitochondrial function and restored energy balance in the ischemic heart by the activation of AMP-activated protein kinase (AMPK) (72). Therefore, FGF21 can be considered as both a predicted biomarker and a potential protective agent against AMI. All these discoveries provide new strategies for the treatment of ischemic arrhythmia. It is necessary to conduct more prospective studies to clarify these issues.

## The role of fibroblast growth factor 21 in acute kidney injury

Acute kidney injury is a clinical condition defined as a rapid decline in renal function. Lots of factors including drugs, infections, and ischemic can lead to the occurrence of AKI (73). The pathophysiology of AKI is characterized by the inflammation response, cell apoptosis, and fibrosis after kidney injury, which may be regulated by multiple molecular regulators, such as adenosine receptors, peptidylarginine deiminase 4, and Toll-like receptors (74). Circulating FGF21 levels were increased in acute and chronic renal dysfunction mice (75, 76), and recombinant FGF21 also showed therapeutic potential in cisplatin-induced kidney injury (75).

The expression of FGF21 and its receptor was increased in renal mesangial cells of diabetic db/db mice (77). FGF21 levels were significantly elevated in patients with AKI based on clinical data (75, 76). Thus, this indicates a theory that FGF21 is associated with AKI and may regulate the process of AKI.

Knockdown of FGF21 increased tubular apoptosis, but supplementation with recombinant FGF21 protected tissue damage and improved kidney function by inhibiting p53 expression in cells and mouse models (75). The FGF21 expression in cisplatin-induced AKI models was significantly elevated according to a recent study (78). Furthermore, FGF21 can decrease apoptotic cells and alleviate cisplatin-induced acute renal injury by upregulated expression of silent information regulator sirtuin 1 (SIRT1) (78). FGF21 inhibited the expression of pro-inflammatory cytokinesis by nuclear factor kB (NF-kB) signaling and suppressed oxidative stress by upregulated superoxide dismutase (SOD) and glutathione (GSH) and decreased the expression of malondialdehyde (MDA) *in vitro* (79). FGF21 may also regulate mitochondrial and oxidative stress (80). Then has the expected protective effects in AKI. Although scholars have increased interest in FGF21 and kidney function in recent years, FGF21 is still not fully understood in terms of its role in kidney development. Evidence between FGF21 and renal function is still limited, so more research will be needed in the future.

## The role of fibroblast growth factor 21 in sepsis and septic shock

The disease of sepsis is characterized by organ dysfunction caused by infection and is considered a life-threatening condition (81). In 2017, the world suffered 48.9 million new sepsis cases, among which up to 11 million patients died of sepsis, and it is responsible for 19.7% of all deaths worldwide (82). Based on the data from the Chinese sepsis epidemiological survey, one-fifth of patients in ICUs of the Chinese mainland suffered from sepsis, and 35.5% of them died within 90 days (83). Although increasing biomarkers related to sepsis have been studied, the ideal diagnostic and prognostic accuracy of biomarkers for sepsis is not yet established (84).

In the early course of sepsis, inflammatory and anti-inflammatory cytokines were increased (85). According to previous studies, FGF21 has played an anti-inflammatory role in sepsis (86). Due to this, it should not come as a surprise that FGF21 levels in the circulation were elevated in both sepsis mouse models and patients with sepsis (16, 86, 87), indicating that this increase may act as a protective mechanism. In addition, increasing FGF21 levels in muscles and adipose tissue might be a contributing factor (86). Circulating FGF21 is liver derived, and it maintains thermoregulation and preserves cardiovascular function during bacterial inflammation, and finally decreased the mortality in endotoxemia (71). Thus, FGF21-deficient mice are more easily to die of endotoxemia, and initiated administration of FGF21 after bacterial inflammation can get a better survival (88).

Besides, FGF21 can predict the prognostic survival for patients with sepsis, and patients with sepsis with FGF21 levels below 3,200 pg/ml had a significantly lower mortality rate than those with levels above 3,200 pg/ml (16). When it comes to predicting 28-day mortality, FGF21 had a sensitivity of 81.3% and a specificity of 89.8%, respectively (16). As for patients with sepsis and ARDS, the expression of FGF21 levels persistently increased until the peak time points of shock and death. Compared with patients who survive, FGF21 levels were nearly four times higher in patients with sepsis and ARDS who did not survive (17). Thus, FGF21 may be a potential biomarker for sepsis patients’ survival prediction. FGF21 is also required for controlling the inflammation of metformin through suppressing pro-inflammatory cytokines and enhancing anti-inflammatory cytokines in rat liver induced by LPS (89). The relationship between increased expression of FGF21 and sepsis may be explained by these findings. Unfortunately, population-related clinical trials with FGF21 or the analogs of FGF21 in the treatment of sepsis have not proceeded. Furthermore, the underlying mechanism of the protective effects of FGF21 remains unclear in sepsis, and it may need more studies.

## The role of fibroblast growth factor 21 in liver failure

Liver failure is one of the most common and deadly clinical syndromes (90). Many factors can cause liver failures, such as hepatitis virus, drug, paracetamol toxicity, and autoimmunity (91). The pathology of liver failure commonly demonstrates extensive hepatic apoptosis and necrosis. However, liver failure often accompanies by systemic inflammation. Currently, few studies have examined FGF21’s role in liver failure.

The FGF21 played a protective role in liver failure in a Kunming mice model induced by D-galactose (92). It reduced liver and histological injury and inhibited hepatocyte oxidative stress by the enhancement of Nrf2-dependent antioxidant capacity and apoptosis through activating PI3K/Akt signaling (92). In acute liver failure induced by acetaminophen (APAP), both the hepatic and circulating expressions of FGF21 were markedly increased within 3 h, which therefore was a feedback signal that protected mice from APAP-induced liver damage by enhancing liver anti-oxidative defenses (93). Therefore, FGF21 could be severed as an early diagnostic and therapeutic factor for APAP-induced acute hepatic injury. Moreover, the absence of FGF21 exacerbated oxidative stress and resulted in severe liver injury and high mortality, and these can be reversed by the replenishment of recombinant FGF21 in mice (93). FGF21 inhibited the hepatotoxicity and mortality caused by APAP by inducing peroxisome proliferator-activated receptor coactivator protein-1α functionally (93).

The serum FGF21 levels after liver transplantation displayed distinct dynamic profiles, the FGF21 levels were low (< 300 pg/ml) within 1 h, and then they had a sharp rise and reached the peak earlier just at 2 h (94). However, serum ALT level reached the highest level at 24 h after reperfusion (94). All these results prove that serum FGF21 has a higher sensitivity than current biomarkers used to detect liver I/R injury (94). Nevertheless, the role of FGF21 that plays in hepatic I/R injury is unclear. The elevation of FGF21 may be an adaptive and protective response to a rapid onset of cell death.

Acute-on-chronic liver failure usually develops in patients with cirrhosis who have acute decompensation and has a high mortality (90). A decrease in serum FGF21 was observed with cirrhosis when the liver synthesis function was decreased, suggesting that a specific behavior of FGF21 may result in cirrhosis (18). In addition, FGF21 had a predictive value in the development of ACLF. However, FGF21 did not show an association with mortality for these patients (18). In contrast, the level of FGF21 was markedly increased when ACLF occurred, and it might reflect the injury degree of the liver (18). According to another study, serum FGF21 expression was predominantly upregulated in patients with severe ICU cirrhosis and had an important diagnostic value in patients with ACLF (20). All these data suggested a fact that FGF21 had an important role in liver failure, but the exact mechanism of FGF21 in ACLF has not been engaged deeply, and the clinical utility also needs further systemic investigation.

## Application prospect of fibroblast growth factor 21

**FIGURE 2:**
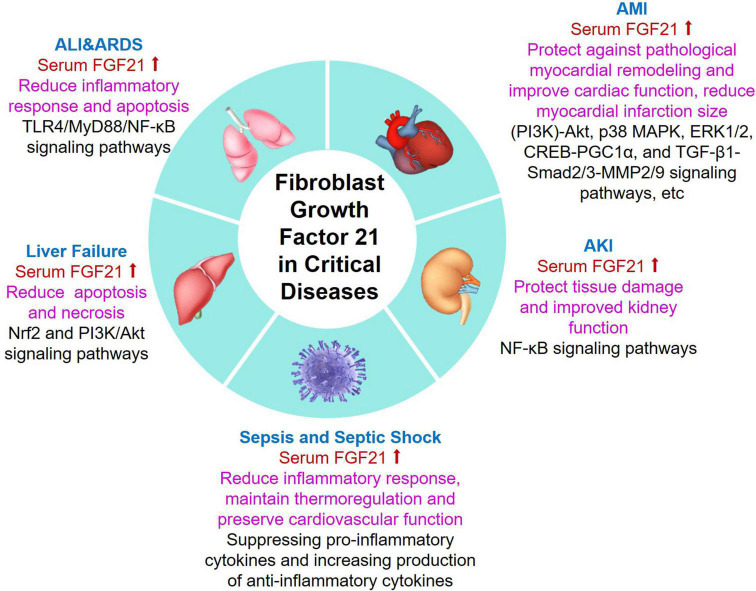
Summary of emerging roles and related mechanisms of FGF21 in critical diseases. Serum FGF21 levels were increased in patients with critical diseases including ALI/ARDS, AMI, AKI, sepsis, and liver failure. Additionally, FGF21 plays different biological functions in critical patients with different underlying mechanisms. ALI, acute lung injury; ARDS, acute respiratory distress syndrome; AMI, acute myocardial injury; AKI, acute kidney injury; FGF21, fibroblast growth factor 21.

In summary, FGF21 is closely associated with critical disease and has potential benefits for acute organ injury, and it plays an important role in critical disease (Figure 2). Since FGF21 does not show significant performance in promoting mitosis in cell lines and mice (51), it is suggested that FGF21 has excellent safety. Despite FGF21 has a variety of benefits, due to its small relative molecular weight (about 20 KD), it is easily degraded by protease *in vivo* and filtered by the glomerulus, and its half-life in plasma is only 0.5–1.5 h (95–97). The clinical route of application of FGF21 has been limited to some extent. However, it is encouraging that in recent years, long-acting FGF21 and its analogs have been gradually developed.

The FGF21-based therapies are relatively new for clinical application, and the extensive biological characteristics of the FGF family have not been fully utilized in the treatment of human diseases. We believe that FGF21 will have great development space in critical diseases and other clinical illnesses in the future.

## Author contributions

FY (1st author): conceptualization, methodology, and writing. LY and FY (3rd author): data curation and writing the original draft. XJ and GW: conception and design of the manuscript. All authors contributed to the article and approved the submitted version.
